# Postgraduate Medical Training in India: Inadequacies and Challenges Faced by Young Medical Graduates

**DOI:** 10.7759/cureus.94053

**Published:** 2025-10-07

**Authors:** Muhammad A Hamid, Zubair Younis, Shahid Mir, Ariz Raza, Nayan Shrivastava, Rishi Raj

**Affiliations:** 1 Trauma and Orthopaedics, East and North Hertfordshire Teaching NHS Trust, Stevenage, GBR; 2 Trauma and Orthopaedics, The Royal Wolverhampton NHS Trust, Wolverhampton, GBR; 3 Trauma and Orthopaedics, Manchester University NHS Foundation Trust, Manchester, GBR; 4 Trauma and Orthopaedics, University Hospitals Birmingham NHS Foundation Trust, Birmingham, GBR; 5 Trauma and Orthopaedics, Altnagelvin Hospital, Londonderry, GBR

**Keywords:** md/ms/dnb, medical graduates, national medical council, post-graduate medical education, specialist training

## Abstract

Postgraduate (PG) medical education is vital for a nation’s healthcare workforce development. Over the past decade, significant reforms have been introduced under the leadership of the National Medical Commission (NMC) and the National Board of Examinations in Medical Sciences (NBEMS), including expansion of training capacity and efforts to standardize assessment through national-level entrance and exit examinations. Despite these advances, the system continues to face formidable challenges. A persistent mismatch between the number of MBBS graduates and available PG seats has created a highly competitive environment, forcing many candidates into repeated examination cycles. Geographical and specialty-based disparities remain stark, with a concentration of training opportunities in some states and oversubscription of high-demand specialties. The rapid growth of medical colleges has not been matched by proportional faculty recruitment, raising concerns about dilution of academic quality and inadequate supervision of trainees. Trainee doctors report excessive workloads, long duty hours, insufficient infrastructure, and limited structured academic support, all of which contribute to burnout, impaired performance, and stress-related morbidity. These pressures are compounded by an alarming rise in workplace violence, with nearly three-quarters of doctors in India reporting exposure to some form of abuse or assault. In addition, frequent policy shifts, including postponements of the National Eligibility cum Entrance Test for Post-graduate training (NEET-PG) and uncertainty around the proposed National Exit Test (NEXT), add to the psychological burden of young doctors. While expansion of PG training capacity is crucial, it must be matched by equitable distribution, faculty development, modern teaching methodologies, and systemic safeguards for trainee welfare. These are essential to safeguard both the quality of PG training and the sustainability of India’s future healthcare workforce. This narrative review was conducted using keyword-based searches in PubMed, Google Scholar, and Scopus, supplemented by official NMC and NBEMS documents, Ministry of Health reports, and credible national news sources. Literature published from 2010 was included if it addressed PG medical education in India. Both peer-reviewed and policy sources were examined narratively to capture trends and highlight systemic challenges.

## Introduction and background

Postgraduate (PG) medical education is a pivotal stage in the training of doctors, shaping the nation’s specialist workforce and directly influencing the quality, accessibility, and equity of healthcare delivery [1]. In India, the system of PG training has undergone rapid transformation in recent years, reflecting both the rising demand for specialist services and the growing pressure to align medical education with global standards. With a population exceeding 1.4 billion and an evolving burden of both communicable and non-communicable diseases, India faces a dual challenge: expanding the number of doctors to meet basic healthcare needs while simultaneously producing specialists capable of delivering advanced care [2].

The regulatory framework for PG training is now governed primarily by the National Medical Commission (NMC), established in 2020 to replace the Medical Council of India (MCI), which was the statutory body responsible for establishing and maintaining the standards of medical education in India. The NMC functions through its Postgraduate Medical Education Board (PGMEB), which is responsible for accreditation of courses, development of competency-based curricula, and implementation of assessment reforms [3,4]. In parallel, the National Board of Examinations in Medical Sciences (NBEMS) conducts high-stakes national entrance and exit examinations, including the National Eligibility-cum-Entrance Test for Postgraduate (NEET-PG) and the National Eligibility-cum-Entrance Test for Super-Speciality (NEET-SS). NBEMS also administers the Diplomate of National Board (DNB) and Doctorate of National Board (DrNB) qualifications, which are considered equivalent to university MD/MS and DM/MCH programs, respectively [5]. These two pathways are standardized PG training programs in India after obtaining a primary medical qualification. These parallel pathways were designed to expand capacity and diversify training opportunities, particularly in private and non-teaching hospitals.

India’s PG training system is one of the largest globally, yet its capacity and quality remain insufficient to meet the growing demands of the healthcare system [6]. As of 2024, over 228,000 Bachelor of Medicine and Bachelor of Surgery (MBBS) graduates competed for approximately 74,000 PG seats, creating a significant undergraduate-to-postgraduate (UG:PG) gap [7].

This review examines the systemic challenges affecting PG medical training in India, drawing on official policy documents, peer-reviewed literature, and government reports. A narrative review methodology was adopted without performing statistical analysis, and keyword-based searches were carried out in PubMed, Google Scholar, and Scopus using various combinations of ‘India’, ‘post-graduate training’, ‘medical training’, ‘NEET-PG’, and ‘NMC’ to identify relevant academic and non-academic works from 2010 onwards. Other sources included the NMC website, NBEMS notifications, and major national newspapers reporting on policy changes.

## Review

Seat shortages and UG:PG gap

The most prominent challenge is the mismatch between the number of MBBS graduates and available PG training positions. As of writing this article (2025), annually held standardized entrance examinations - the National Eligibility cum Entrance Test (NEET) - determine admissions to medical college, specialist training, and fellowships [8]. In 2024, 228,540 candidates sat for the NEET-PG examination for approximately 74,000 seats [9]. The government is also in the process of upgrading 157 district-level hospitals to medical colleges, out of which 131 are presently functional [7]. Despite this, there still would be a considerable backlog of qualified medical practitioners unable to progress into specialty training due to the sheer volume of eligible candidates and the dearth of training seats. This results in a UG:PG ratio of 3:1, which creates intense competition, leading many candidates to spend years preparing for entrance examinations or to seek opportunities abroad.

Geographic and specialty distribution inequities

PG seats are concentrated in southern and urban states, leaving rural and underserved regions with fewer training opportunities [10]. Over the past decade, there has been a considerable increase in the number of medical colleges in India. According to the Ministry of Health and Family Welfare, there has been an 82% rise in the number of medical colleges, from 387 in 2013 to 704 in 2023. There has also been a 117% increase in PG training positions from 31,185 in 2013 to 67,902 in 2023. This has been accompanied by a corresponding rise in the number of undergraduate (MBBS) seats as well, from 51,348 to 107,948 (110% increase) [11]. The geographical distribution of newly set-up medical colleges is skewed as well, with two-thirds of newly established medical colleges based in the southern states, serving just one-third of the country’s population [10]. There is also a dearth of training opportunities available in primary care. Despite statutory recognition of family medicine in the NMC Act 2019 and its importance for strengthening primary care, training opportunities remain inadequate [12-14]. Also, many PG training posts remain undersubscribed, leading to the NMC lowering the cut-off marks required to qualify for the examination in subsequent matching rounds [15]. This has led to concerns that such measures undermine academic rigour and lead to lowering the quality of PG medical training [16]. Even super-speciality medical seats like cardiology, nephrology, gastroenterology, and cardiac surgery positions in a number of institutions remained vacant, and the NBEMS had to reduce the qualifying percentile required to fill these positions [17].

Faculty shortages and training standards

In order to meet growing demand, there has been a rapid expansion of medical colleges over the past decade. Unfortunately, this rapid expansion has not been matched by the recruitment of adequately qualified teaching staff. The government also aims to add 75,000 more medical seats over the next five years as part of its plan to boost the country’s health infrastructure [18]. In response, the National Medical Council (NMC) has relaxed faculty qualification requirements, permitting senior non-teaching specialists to be appointed as assistant professors and accrediting 220-bed hospitals as teaching centres [19]. This has led to concerns that these measures may dilute academic standards and compromise medical education [20]. They also limit adequate supervision of trainees, focus on quality research, and ensure delivery of competency-based curricula.

In addition to the disparity of numbers, there are significant institutional problems facing PG medical training. Multiple reports highlight excessive working hours - often exceeding 30 hours continuously, alongside inadequate facilities and support for training residents [21,22]. Such conditions contribute to burnout, depression, and impaired clinical performance. Absence of simulation laboratories and adequate training spaces is one of the infrastructural deficits exacerbating these challenges. This combination of high workload and structural inadequacies leads to poor performance and increased tendency to make mistakes, which can often be life-threatening [23]. A survey of 274 PG trainees revealed stark imbalances in the provision of specialist training and academics. A total of 19.8% trainees reported absent supervision by faculty during their training program, and 20% trainees reported minimal to no academic activities organized by their respective departments. A combination of high workload, minimal supervision, and limited exposure to academics also leads to high stress levels in trainee physicians, impacting their well-being as well as clinical performance [24].

In strengthening PG medical training, investment in structured simulation-based education and formal mentorship programmes represents a practical way forward. Simulation laboratories allow trainees to practise complex procedures and emergency scenarios in a controlled environment, reducing reliance on service-based learning alone and improving patient safety [25]. Equally, structured mentorship - pairing junior residents with experienced faculty - can provide guidance in clinical decision-making, research, and professional development, while also helping to buffer the psychological strain of long hours and high workload [26]. Integrating these elements into PG curricula would not only address gaps in infrastructure but also promote more consistent, competency-based training outcomes across institutions.

Violence against doctors

Violence against doctors in India has become a pervasive occupational hazard that undermines clinical care, erodes trust in the health system, and accelerates burnout and attrition among healthcare workers [27]. PG specialist trainees, being at the frontline in critical areas, are often the subject of this violence. A systematic survey by the Indian Medical Association in 2018 found that approximately 75% of doctors in India have experienced some form of workplace violence, with emergency services being the most common setting and relatives of patients involved in around 70% of incidents [28]. Episodes range from verbal abuse, intimidation, and social-media harassment to physical assault, mob vandalism, and threats of medico-legal entanglement, with the emergency department, intensive care units, and obstetrics being disproportionately affected [29,30]. The drivers are multifactorial and mutually reinforcing: chronic underfunding of public facilities results in overcrowding, long waiting times, and resource constraints; rising out-of-pocket costs in the private sector magnify financial distress and unrealistic expectations; gaps in communication, adverse outcomes in complex surgeries, or delays in bed allocation can be misconstrued as negligence; and sensationalist reporting or misinformation on social platforms can rapidly mobilize crowds before facts are established. Structural weaknesses compound the problem - limited triage segregation, inadequate visitor management, and insufficient security presence leave frontline clinicians exposed, while inconsistent medico-legal protocols and slow grievance redressal mechanisms fuel perceptions of impunity. The consequences are profound: clinicians adapt by practicing defensive medicine, avoiding high-risk cases, or migrating to perceived safer specialties and geographies, which in turn worsens access for critically ill patients and amplifies inequities [31]. The psychological toll - moral injury, hypervigilance, sleep disturbance, and demoralization - feeds attrition and diminishes teaching and research culture in already stretched institutions. Although several states have enacted healthcare-specific protection laws and the Epidemic Diseases (Amendment) Act, 2000, temporarily strengthened penalties for violence against healthcare workers during pandemics, enforcement remains uneven, convictions are rare, and many incidents go unreported due to fear of retaliation or administrative fatigue [32]. Sustainable prevention requires a layered approach: hardening of hospital infrastructure with controlled entry points, CCTV coverage of high-risk zones, panic alarms, and trained security; operational redesign that improves flow - fast-track triage, clear bed dashboards, family-counselling bays, and transparent display of waiting times and costs; mandatory communication and de-escalation training for all cadres, with scripts for breaking bad news and standardized consent processes; 24/7 grievance desks and rapid incident-review protocols that involve clinical leaders, legal counsel, and patient representatives; and formal liaison with local law enforcement including on-call police presence during surge hours [33]. At the policy level, a centrally enacted, uniformly enforceable law with non-bailable provisions, time-bound investigation, and compensation for property damage would reduce jurisdictional ambiguity, while medico-legal reform that clarifies standards for negligence and protects good-faith clinical judgment could curb opportunistic litigation. Finally, public education campaigns that explain the limits of medicine, promote realistic expectations, and humanize clinicians - paired with investments to expand critical-care capacity, emergency transport, and health insurance coverage - address the upstream pressures that often precipitate conflict [34]. Without such systemic measures, sporadic crackdowns will not suffice; protecting doctors is inseparable from protecting patients, because safe caregivers are a prerequisite for safe care.

Policy instability and exam uncertainty

Policy changes surrounding the selection process for PG medical training in India have repeatedly complicated the career trajectories of young doctors and contributed to widespread uncertainty. At the centre of this is the NEET-PG, the sole entrance pathway for specialist training courses, which has been subject to frequent alterations in timing, format, counselling rules, and cut-off criteria. Over the past decade, postponements of NEET-PG examinations - sometimes announced at the last minute - have disrupted academic calendars and forced graduates into prolonged cycles of preparation, delaying specialist training and creating mental stress among thousands of aspirants [35,36]. The situation has been further complicated by ongoing debates about replacing NEET-PG with the National Exit Test (NEXT), proposed as a common licensure and PG entrance examination under the National Medical Commission Act 2019 [37]. Uncertainty over the rollout of NEXT has generated confusion, with successive batches of MBBS graduates unsure whether they will sit for NEET-PG, NEXT, or an altered hybrid model [38]. Counselling procedures have also been a flashpoint: delays in seat allotment, sudden changes in reservation policies, and state-level deviations from national quota norms have triggered legal disputes and even Supreme Court intervention, further prolonging the admission process [39]. Even super-specialty admissions under NEET-SS, as outlined earlier, have witnessed abrupt changes in eligibility criteria and percentile thresholds, leading to vacancies in some disciplines despite high demand in others. Collectively, these policy fluctuations erode the predictability of the training pathway, forcing young doctors to divert energy from academic preparation toward navigating administrative complexity. Many candidates spend years in repeated examination cycles, often balancing clinical duties with extensive study requirements, which exacerbates burnout and affects mental health [40].

## Conclusions

PG medical education in India has expanded significantly, yet persistent challenges such as seat shortages, geographic and specialty imbalances, faculty deficits, heavy workloads, workplace violence, and policy instability continue to compromise training quality. To address these, reforms should focus on equitable distribution of training opportunities, strengthening faculty recruitment and mentorship, investing in infrastructure, including simulation-based education, ensuring trainee welfare and workplace safety, and stabilizing admission and examination policies. By aligning expansion with quality safeguards and workforce equity, India can build a PG training system that produces skilled specialists while meeting the country’s diverse healthcare needs.
